# A novel proposed classification system for rock slope stability assessment

**DOI:** 10.1038/s41598-024-58772-7

**Published:** 2024-05-14

**Authors:** Amit Jaiswal, A. K. Verma, T. N. Singh

**Affiliations:** Department of Civil and Environmental Engineering, Indian Institute of Technology Patna, Patna, 801106 India

**Keywords:** Rock mass classification, Rock slope, Himalayan rock, Slope stablity, Civil engineering, Natural hazards

## Abstract

The present study introduces “rock slope instability score (RSIS)” a novel classification system for assessing rock slope stability. It takes into account geological and geotechnical parameters, as well as the impact of human activities and triggering parameters, which have become more frequent due to climate change and few of them have been ignored in existing classifications. The study focuses on rock slopes of various lithologies from the Indian Himalayas. The development of this new classification system is based on the examination of 81 different rock slopes from various states of the Indian Himalayas. Extensive field surveys, rock sampling, geotechnical laboratory tests, and ground measurements have been conducted at the various slope sites to establish a comprehensive scoring system for the stability assessment. The distributions of weightage to each parameter have been considered, corresponding to its degree of impact in causing slope instability. Sensitivity analysis of all defined parameters of RSIS system has revealed that the majority of the parameters exhibit a strong positive correlation, with Pearson correlation coefficients ranging from 0.74 to 0.61. However, two parameters, namely discontinuity dip and the relationship between slope & discontinuity direction, gives moderate relationship with correlation coefficient values of 0.48 and 0.41, respectively. To avoid any designer biasness in the system, several individuals gathered data set at different times. The proposed classification system has demonstrated a strong correlation with the actual slope condition, and it is quite promising. The outcome of RSIS classification for studied 81 slopes classified 2 slopes under stable condition, 21 slopes as partially stable, 44 as unstable, and 14 as completely unstable.

## Introduction

The accurate assessment of slope stability is a crucial task in the construction and design of various civil engineering projects, including dams, highways, open pits, and other excavations Mahdiyar et al.^1^. Geotechnical engineers and geologists frequently rely on analytical and empirical methods to assess the stability condition of soil or rock slopes, taking into account design parameters and engineering properties Asteris et al.^2^. The key objective of slope stability analysis is to minimise its chance of failure and determine economic design^3^. Numerical techniques like finite element method (FEM) Hutton^4^, discrete element method (DEM) Cundal et al.^5,6^, etc. have a limitation of real-time estimation of the stability of road cut slopes as the excavation is usually too fast for these analysis. Rock slope cutting have similarity with large span rock caverns in terms of stress changes which are sufficiently stationary. To overcome the above limitations, geotechnical professionals and researchers have devised empirical methods (rock mass classification) for quickly quantifying and assessing slope stability conditions Barton and Bar^7^. Ritter^8^ for the first time tried to formalize an empirical method for tunnel design, along with support requirements. Several engineering rock mass classification schemes have been proposed from different parts of the world post Ritter^8^ classification, but only few have gained recognition and are widely used for categorization of rock masses. Terzaghi^9^ and Stiniv^10^ classifications have provided the foundation for the two most relevant modern geomechanics classifications, i.e., rock mass rating (RMR) Bieniawski^11^, and the rock tunneling quality index, Q-system Barton and Barton Grimstad^12,13^. The efficacy of the rating system as a tool for categorizing rock formations and slopes was promptly realized, leading to the development of several novel classification systems. These systems were founded on the rating concept initially proposed by Wickham et al.^14^ for the rock structure rating (RSR) system. Deere^15^ introduced the rock quality designation index (RQD) to quantitatively evaluate rock mass quality from drill cores for sound rocks. Some of the modified versions of RMR classification system like modified basic RMR (MBR) Cummings et al. and Kendorski et al.^16,17^, RMR_89_ Bieniawski^18^, Rock Mass Index (RMi) Palmstro¨m^19^, Modified RMR (M-RMR) U¨ nal et al. and U¨ nal^20,21^, have been proposed with the span of time. RMR_89_ has been a significant application in the field of rock engineering for several decades, and some researchers Beemer and Worrells^22^ have conducted simulation experiments where RMR tasks were performed during analogue Mars missions. A classification system for rock slopes known as slope mass rating (SMR) was proposed by Romana^23^ as an addition to the RMR system. However, researchers such as Goel and Singh^24^ have highlighted its limitations in the case of tightly joined rock masses and large-scale rock slopes. The mining rock mass rating system Haines and Terbrugge^25^ has also been used to assess rock slope stability. Geological strength index (GSI), developed by Hoek et al.^26^, and has been modified several times. Hoek and Brown^27^ created the GSI based on the visual impression of the rock mass structure. Sonmez and Ulusay^28^ modified this classification system to provide a more quantitative numerical basis for evaluating the GSI. Continuous slope mass rating (CoSMR) and Chinese slope mass rating (CSMR) are revised versions of the SMR system developed by Tomás et al.^29^ and Chen^30^, respectively. These systems, along with other classification methods like SSR Taheri et al.^31^ and Q-slope Barton and Bar^7^ were designed to provide quantitative assessments of rock mass properties, which were previously described qualitatively in geological reports. All these above-mentioned systems were originally developed to design tunnels and underground structures.

Over the last few decades, numerous qualitative empirical techniques for evaluating the stability of rock slopes have been developed and documented in scientific literature. Each classification was designed for a specific purpose, especially for underground excavation, which has certain limitations. However, none of the classifications available today can comprehensively assess the condition of a rock slope based on the combined influence of geomechanical parameters, geological structure, seismicity, annual rainfall and anthropogenic effect that may cause instability. This new classification system considers different quantitative assessment methods for different intensities of seismicity and different environmental effect while calculating the rock slope stability score. Considering the limitations of all the existing empirical classification systems, a new rock slope classification system called the rock slope instability score (RSIS) has been proposed. The RSIS approach combines the most relevant factors that influence rock slope stability, including geomechanical factors, geological structure and seismic parameters, environmental factors, and anthropogenic factors and offers a quantitative measure for evaluating the instability in the rock slopes. However, main limitation of RSIS is that it cannot assess the slope condition based on monthly or day rainfall which is one of the essential components of real time monitoring rock slope stability. Furthermore, insufficient consideration of the percentages of deviation in defining the range of different parameters may have led to the incorrect evaluation of few slopes which will be addressed in future revised version of RSIS system.

The novel classification system presented in this research paper aims to evaluate the stability of rock slopes by considering the increasing frequency of extreme weather events linked to climate change and the enhanced capabilities of instruments in detecting even minor changes in factors affecting rock slope stability. This system accounts for the geotechnical and geological parameters of rock slopes, alongside the influences of triggering factors, environmental parameters, and human activities. The study concentrates on rock slopes in the Indian Himalayas and is based on the analysis of 81 diverse rock slopes from various states in the Himalayan region. Extensive field surveys, rock sampling, geotechnical laboratory tests, and ground measurements were carried out to establish a comprehensive scoring system for stability assessment. The determination of the weights assigned to each parameter is based on their respective impact in causing slope instability.

## Methodology and case studies

The relevant information required for this new classification system has been gathered from various locations present within the Indian Himalayan region. Table 1 shows the number of slopes taken from different Himalayan states of India. Rainfall data was sourced from the respective state meteorological department, while seismic data was taken from the seismic zonation map of India. Other sufficient relevant information and field data were collected from all 81 natural and man-made rock slopes during fieldwork Ghosh et al.^32^, Siddique et al.^33^, Sardana et al.^34^ Khanna and Dubey^35^, Verma et al.^36^, Kumar et al.^37^, Dutta et al.^38^, Jaiswal et al.^39^. Rock samples collected from the field were tested in the laboratory according to ASTM standard: D2938 to determine the uniaxial compressive strength (UCS). The lithologies of the analysed rock slopes include all three types of rock: igneous, metamorphic, and sedimentary from different regions of Himalaya representing spatially variable dataset. The majority of rock types taken from Jammu and Kashmir are slates and phyllite, while from Himachal Pradesh are quartzite and schist, whereas from Uttarakhand limestone, metabasic, and quartzite were considered. Additionally from the Sikkim region majority of considered rock types are phyllite and gneiss, whereas from Mizoram, are shale and sandstones. All these rock types exhibit varying degrees of weathering, ranging from fresh to highly weathered condition. Due to spatially distributed dataset there are variations in the slope geometry, shape, and boundary Ghaderi et al.^40^. The slope angle varies from 15° to 90°, and slope aspects cover the complete 360° (i.e., all four quadrants).

**Table 1:** State-wise number of studied slopes.

S. No.	Region	State	Number of Slope {slope id}
1	North Western Himalaya	Himachal Pradesh	10 {HS-1 to HS1-10}
2	North Western Himalaya	Uttarakhand	29 {RD-1 to RD-5; DA-1 to DA-6; RL-1 to RL18}
3	North Western Himalaya	Jammu & Kashmir	14
			{JL-1 to JL-14}
4	North Eastern Himalaya	Mizoram	13
			{ML-1 to ML-13}
5	North Eastern Himalaya	Sikkim	15
			{SL-1 to Sl-15}

## Concept of newly proposed rock slope classification system

Most of the civil and mining engineering industries require estimation of roc slope stability for different projects at its various stages like feasibility, excavation and operation. It is then imperative to quickly and reliably estimate the design parameters of required for existing slopes. Most rock mass classification had been first developed for tunnel design and other underground structures. Slope mass rating (SMR) Romana^23^ was based on RMR, which was developed initially for underground excavation. Similarly, Q slope Barton and Bar^7^ proposed is based on Barton’s Q system Barton^12^, which was originally developed based on case studies of underground excavation. The above slope classification lacked case studies from the surface. The accuracy and resolution of parameter ranges in the case studies of underground excavation, like discontinuity spacing, will not give accurate and reliable outcomes if applied to a slope of several hundred meters.

Similarly, the application of GSI (1995, 1997, 2000, & 2002) to slope stability design is conditional, which suffers from analogous limitations as mentioned above. GSI (1995; 1997) is founded on the assumption that rock masses behave isotropically, which is not supported by factual evidence. Additionally, GSI (2002) needs to be improved when assessing rock masses affected by tectonic disturbances. Furthermore, despite the significant impact of rainfall on slope stability, particularly through the infiltration of water into exposed discontinuities and the subsequent development of hydrostatic pressures reducing the shear strength, existing rock mass classification systems do not sufficiently account for this effect. Therefore, a new rock slope classification system, “Rock Slope Instability Score (RSIS)” has been developed to overcome this constraint and consider all critical parameters influencing slope stability. During the development of this new classification system, a detailed study of the globally accepted rock mass classification system and landslide hazard zonation system proposed by Anbalagan^41^, Anbalagan et al.^42^ has been done.

The RSIS system divides the rock slope characteristics into four main categories: rock characteristics (RC), kinematic condition (KC), triggering effect (TE), and anthropogenic effect (AE) (Table 2). Each of these four categories is further divided into subcategories, which are discussed in detail in the following sections. The cumulative score of all parameters from the above-mentioned categories indicates the degree of instability of any slope (Eq. 1). A higher score indicates unfavorable conditions and a comparatively higher risk of failure. Conversely, a lower cumulative score signifies a lower risk of failure. The scoring system varies from 0 (minimum value) to 100 (maximum value). The score assigned to an individual category or parameter is based on its influence in causing instability. The higher the scoring value given to any category or parameter, the higher is the influence of those category or parameter.

**Table 2:** Classification factors and their scores for rock slope instability score (RSIS) (see Table 3).

Factors	Categories (Percentage)	Value range										Maximum score
*Rock slope instability score (RSIS)*												
Rock strength factor (RSF)	Rock characteristics (RC) (30%)											15
Score												
Rock class		Class -1		Class -2		Class -3		Class-4		Class -5		15
Score		2.5–3.75		4.5–6.75		6.5 -9.75		8–12		10–15		
Slope angle	Kinematic condition (KC) (40%)			< 30		30–55		55–70		> 70		5
Score				1		2.5		4		5		
Discontinuity dip				< 30		30–55		55–70		> 70		5
Score				1		2.5		4		5		
Slope aspect		North (355°–5°)		NE/NW/E (85°–95°)/W (265°–275°)				SW(220°–230°)/SE(130°–140°)		South (175°–185°)		10
Score		3		5				7.5		10		
Slope height		< 45 m		45-70 m		70-100 m		100-125 m		> 125 m		10
Score		2		4.5		6.5		8		10		
Slope & Discontinuity direction relation(joint-slope)		> 20		20–10		10–5		< 5				5
Score		1		2.5		4		5				
Slope angle & Discontinuity dip relation (joint-slope)		> 10		10–0		0-to -10		< -10				5
Score		1		2.5		4		5				
Seismic force (Zone)/ Zone factor	Triggering effect (EC) (20%)	0		Zone 2/0.10 g		Zone 3/0.16 g		Zone 4/0.24 g		Zone 5/0.36 g		10
Score		0		2		4.5		8		10		
Annual Rainfall (mm)		< 500		501–1000		1001–1500		1501–2000		> 2000		10
Score		2		4		6.5		8		10		
Slope Excavation Method	Anthropogenic effect (AE) (10%)	Natural		Mechanical		Pre-splitting		Normal blasting		Poor-blasting		10
Score		2		3		5		8		10		
*Rock slope instability score (RSIS) (100)*												
Stability Condition		Not exist				Stable		Partially stable		Unstable		Complete unstable
Scoring Range		< 18.5				18.5–45		45.1–60		60.1–75		> 75

**Figure 1:**
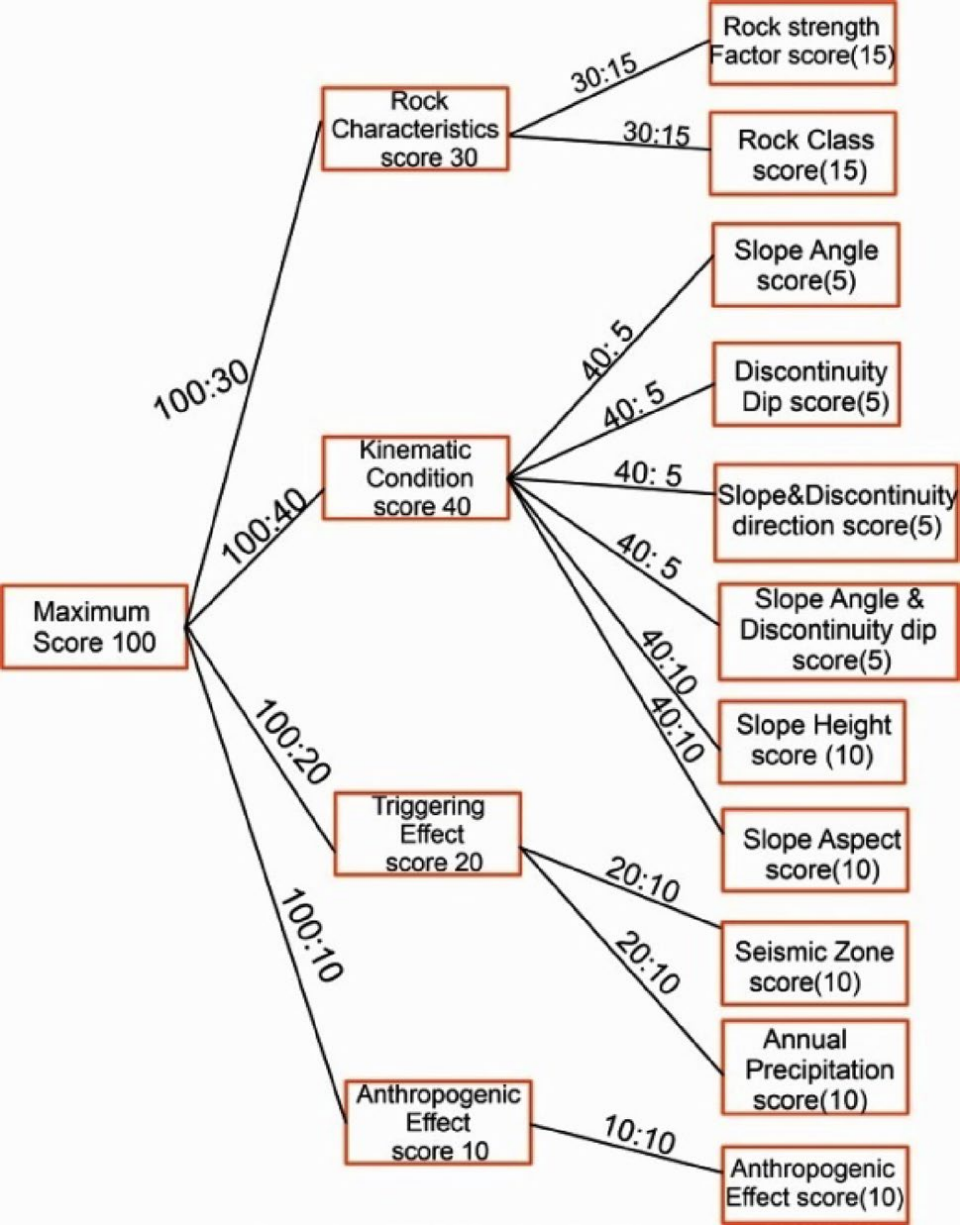
Division of weighting of each factor.

The weightage distribution of each factor corresponds to its susceptibility to potential hazards and to the degree of influence of individual factors in causing slope instability (Fig. 1). Precise weightage assignment to each parameter has been calibrated using a trial and error procedure. This was based on internal expert opinion and knowledge gained in rock mechanics & rock engineering on several case studies performed on Himalayan slopes. Table 1 shows the locations of various slopes across the Himalayas, including widespread rock types used for calibration between expert opinion and the proposed classification system. The first category, “rock characterization” includes rock strength factors and rock type & its degree of weathering, which has been assigned a weightage of 30%. This particular category represents the strength of intact rock, rock type & its weathered condition along with groundwater condition. The most significant weight of 40% is assigned to the “kinematic condition” (slope angle, slope height, slope aspect, discontinuity angle, and relation between discontinuity & slope) category, as it signifies substantial hazard potential in a rock slope. This category is an indicator of the quantity of potential rock blocks generated in a rock slope. The third category is known as “triggering effect” carrying a weightage of 20%. This category indicates the influence of dynamically changing factors in causing slope instability like seismic force & annual rainfall. The last category, “anthropogenic effect” is assigned to human activities like infrastructure development in hilly regions, deforestation, vibrations due to vehicle movements etc. As all these activities significantly influence slope instability, these factors have been assigned a weightage of 10%.

The final score value is as follows:

1 $$RSIS={\mathrm{RC}}+{\mathrm{KC}}+{\mathrm{TE}}+{\mathrm{AE}}$$

where RC is rock characterization; KC is kinematic condition; TE is triggering effect & AE is anthropogenic effect.

Table 2 describes the detailed weightage assigned to individual parameters. The RSIS system allows for a standardized and consistent evaluation of rock slopes, resulting in rock slope management that considers geological, geotechnical, and geomorphological properties along with the impact of human-induced slope instability.

### Rock characteristics (RC) and its scoring

#### Rockmass strength factors (RSF)

RSF has been calculated using Table 3, which includes a summation of scoring values for three different categories:

(i) Strength score, i.e., intact rock strength (Uniaxial compressive strength/ point load),
(ii) Discontinuity ambiance i.e. discontinuity concentration & discontinuity condition);
(iii) Groundwater setting

**Table 3:** Rock discontinuity description for assessment of slope instability.

Rockmass strength factors (RSF)								
Strength Score	Point load (Mpa)	> 10	10–4	4–2	2–1	Low range UCS preferred		
	UCS (Mpa)	> 250	250–100	100–50	50–25	< 25	5–1	< 1
	Score	0.45	0.9	1.2	1.4	1.5	1.8	2
Discontinuity ambience	Concentration	> 2 m	2–0.6 m	600-200 mm	200-60 mm	< 60 mm		
	Score	1.2	2.45	3.5	5.6	7		
	Condition	Persistence	< 1 m	1—3 m	3—10 m	10—20 m	> 20 m	
		Score	0.18	0.4	0.67	0.72	0.8	
		Aperture	None	< 0.1 mm	0.1—1.0 mm	1—5 mm	> 5 mm	
		Score	0.18	0.35	0.62	0.72	0.8	
		Roughness	Very rough	Rough	Slightly rough	Smooth	Slickensided	
		Score	0.18	0.4	0.67	0.72	0.8	
		Infilling	None	Hard filling < 5 mm	Hard filling > 5 mm	Soft filling < 5 mm	Soft filling > 5 mm	
		Score	0.18	0.35	0.62	0.62	0.8	
		Weathering	Unweathered	Slightly weathered	Moderately weathered	Highly weathered	Decomposed	
		Score	0.18	0.4	0.62	0.72	0.8	
Groundwater setting		Completely Dry	Damp	Wet	Dripping	Flowing		
GW level /Slope height ratio		0	0.25	0.5	0.75	1		
	Score	0.45	0.75	1.1	1.5	2		

All the parameters used in the RSF can be evaluated in the field itself, which aids in rock characterization. The cumulative score of these three factors varies on a scale of 0–15 in this new classification system (Table 2).

**Figure 2:**
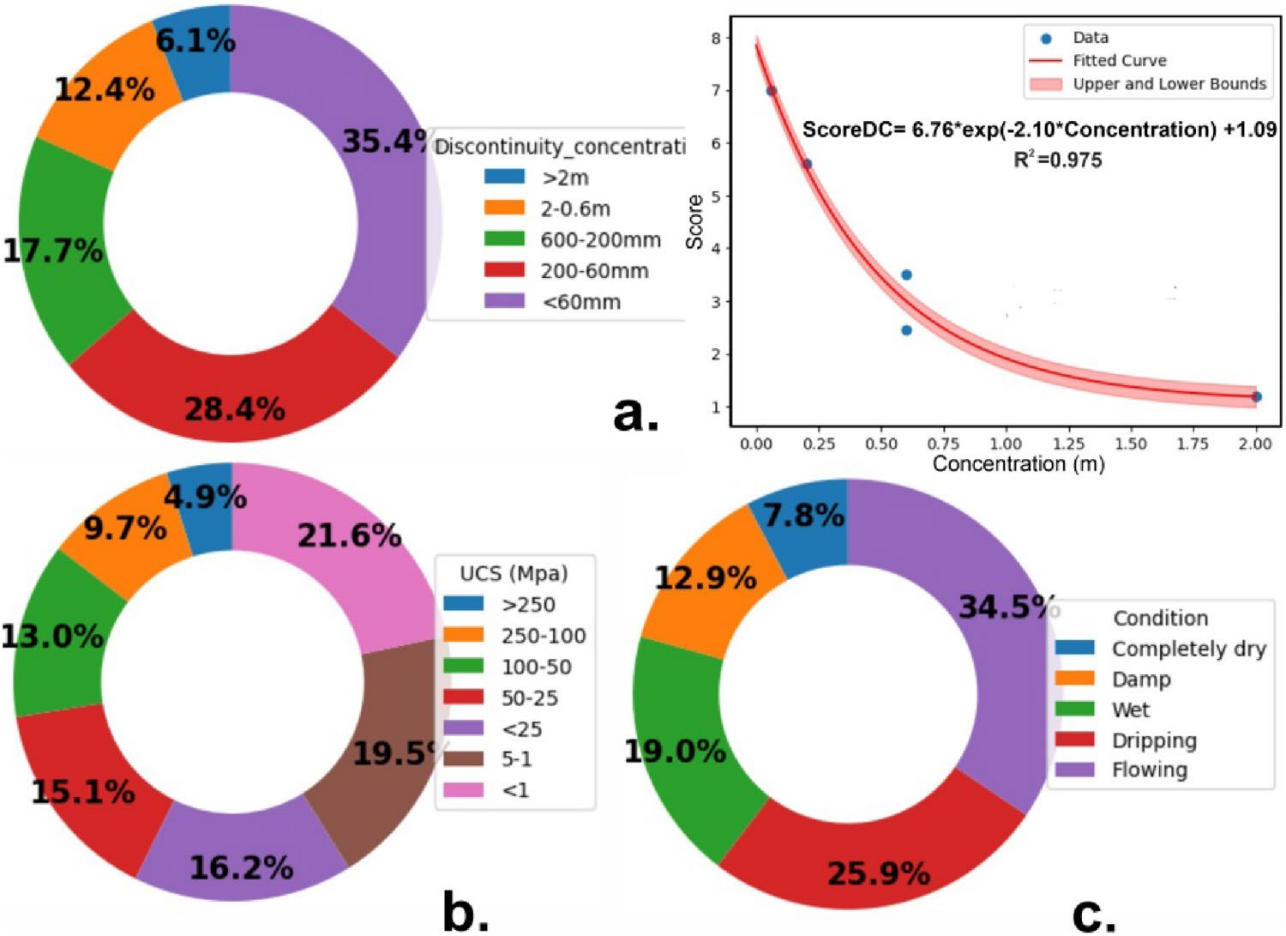
Percentage of weightage assigned by previous researcher (**a**) Discontinuity spacing, (**b**) UCS, (**c**) condition of discontinuity.

The RSF relies on expert’s observations and experience, which can lead to over or underestimation of rock mass conditions. However, the new classification system considers the RSF system as one of the parameters because it includes properties such as intact rock strength, discontinuity spacing, and discontinuity condition, which are crucial factors for assessing slope stability. It also considers groundwater as a factor that was used by only a few classification systems despite its significant influence on the stability of rock slopes. Figure 2 presents the quantified scores of distinct factors computed based on the weightage coefficients assigned by previous researchers who incorporated these parameters within their classification systems. For example, discontinuity concentration of less than 60 mm has contributed to the maximum score value in the total score of discontinuity concentration parameter. This is in accordance with the field observation where rock slopes with closely spaced discontinuity have more chances of instability.

**Figure 3:**
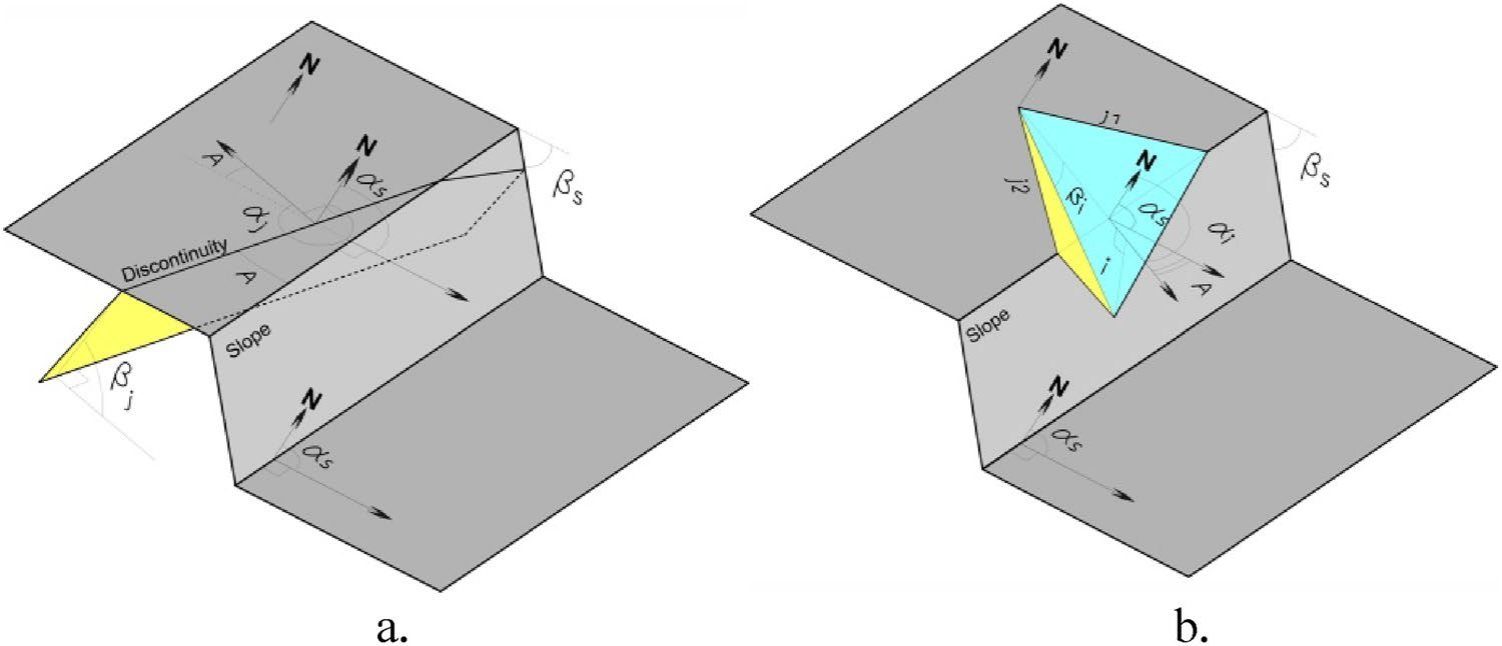
(**a**) Angular relationship between the strike of slope and strike of discontinuity (**b**) Angular relationship between slope angle and discontinuity dip. (adopted after Pastor et al., 2019^43^).

Discontinuity concentration in meters is related to score in an exponential manner, a continuous function given below equation may be used:

2 $$Score = 6.76{e}^{-2.1c}+1.09$$

where c is discontinuity concentration.

#### Rock class

The process of weathering of any particular lithology is contingent upon its composition, which in turn affects the stability of slopes. The rock class is a vital parameter in determining slope stability, leading to the assignment of a maximum scoring value of 15 in the proposed classification system. Rocks have been categorized into five classes based on their chemical composition and susceptibility to weathering. From the perspective of geoengineering, one of the most important parameter for efficiently assessing the stability of slope is the determination depth to bedrock (DTB) Abbaszadeh Shahri et al.^44^. During the development of erosion models, it is imperative to consistently identify the uncertainty and sensitivity of system performance arising from any deviations from the projected input data Asheghi et al.^45^. Scores based on the field condition of rock mass can be calculated with Eqs. 3, 4 and 5. Additionally, for slopes composed of each of these five rock classes, the scoring method specifies two correction values that must be multiplied by the respective score of fresh rock, based on the degree of weathering it has undergone (Table 4).

**Table 4:** Rock classes described for assessment of slope instability.

Rock type	Rock name	Fresh	C_1_ (S-M weathered)	C_2_ (Highly weathered)	Slightly to moderately weathered	Highly weathered
Class-1	Quartzite, Igneous felsic volcanic, Igneous Granite, Marble, dolerite, migmitites	2.5	1.25	1.5	3.125	3.75
Class-2	Limestone, dolomite, Granodiorite, diorite, gabbro, Sedimentary Anhydrite and gypsum, igneous tuff, metamorphic gneiss	4.5	1.25	1.5	5.625	6.75
Class-3	Sedimentary claystone and marl, conglomerate, well cemented and compacted (siltstone and sandstone, mudstone, greywacke), metamorphic Hornfels, mafic volcanic, slaty quartzite,	6.5	1.25	1.5	8.125	9.75
Class-4	Sedimentary Breccia, shale, poorly cemented and compacted (greywacke, sandstone, siltstone, mudstone)	8	1.25	1.5	10	12
Class-5	Metamorphic, Slate, Phyllite Schists and Mylonites	10	1.25	1.5	12.5	15

3 $$Slightly/Moderately\,weathered\,condition\,score=1.25\times Fresh\,rock\,condition\,score$$

4 $$Highly\,weathered\,condition\,score=1.5\times Fresh\,rock\,condition\,score$$

5 $$Rock\,Characteristics \left(RC\right)= \mathrm{RSF\,score}+\mathrm{Rock\,class\,score}$$

### Kinematic condition (KC) and its scoring

The kinematic condition of rock slopes encompasses various parameters such as slope angle, slope height, slope aspect, discontinuity dip, and geometrical relationship between slopes & discontinuities. This relationship considers the direction of the slope in relation to the discontinuity direction and the relation between the slope angle & discontinuity dip.

An increase in values for slope angle, slope height, and discontinuity dip has an adverse effect on slope stability. Therefore, an increase in these parameters is negatively correlated with stability, and the assigned score values also increase accordingly. The slope aspect is a significant and new parameter influencing slope stability. Various studies suggest that south-facing slopes experience greater temperature variations compared to north-facing slopes Flatland^46^, Mazzoccola and Hudson^47^, Branson^48^ and Watters^49^. North-facing slopes, being mostly shaded throughout the day, experience minimal temperature fluctuations, making them less susceptible to slope instability. East, west, northeast, and northwest-facing slopes receive some sunlight during the day and are the third most vulnerable for instability. Southeast and southwest-facing slopes receive the most sunlight and are ranked as the second most vulnerable. South-facing slopes, due to significant temperature changes, are considered the most vulnerable to slope instability (Table 2).

Also, the geometrical relationship between the orientation of slopes and discontinuities has a significant influence on the stability of rock slopes. Two types of relationships (Fig. 3a, b) are defined.

- Slope & discontinuity direction relation
- Slope angle & discontinuity dip relation

The higher the parallelism between these two relationships, the higher the probability of failure. As a result, higher scores are assigned with an increase in parallelism (Table 2). Equation 6 can be used to calculate the kinematic condition score.

6 $$KC=SA score+SH\,score+SAs\,score+DD score+relation\,of\,slope \& discontinuity\,score$$

where KC Kinematic condition, SA Slope angle, SH Slope height, SAs Slope Aspect, DD Discontinuity dip.

### Triggering effect (TE) and its scoring

#### Seismic force

**Figure 4:**
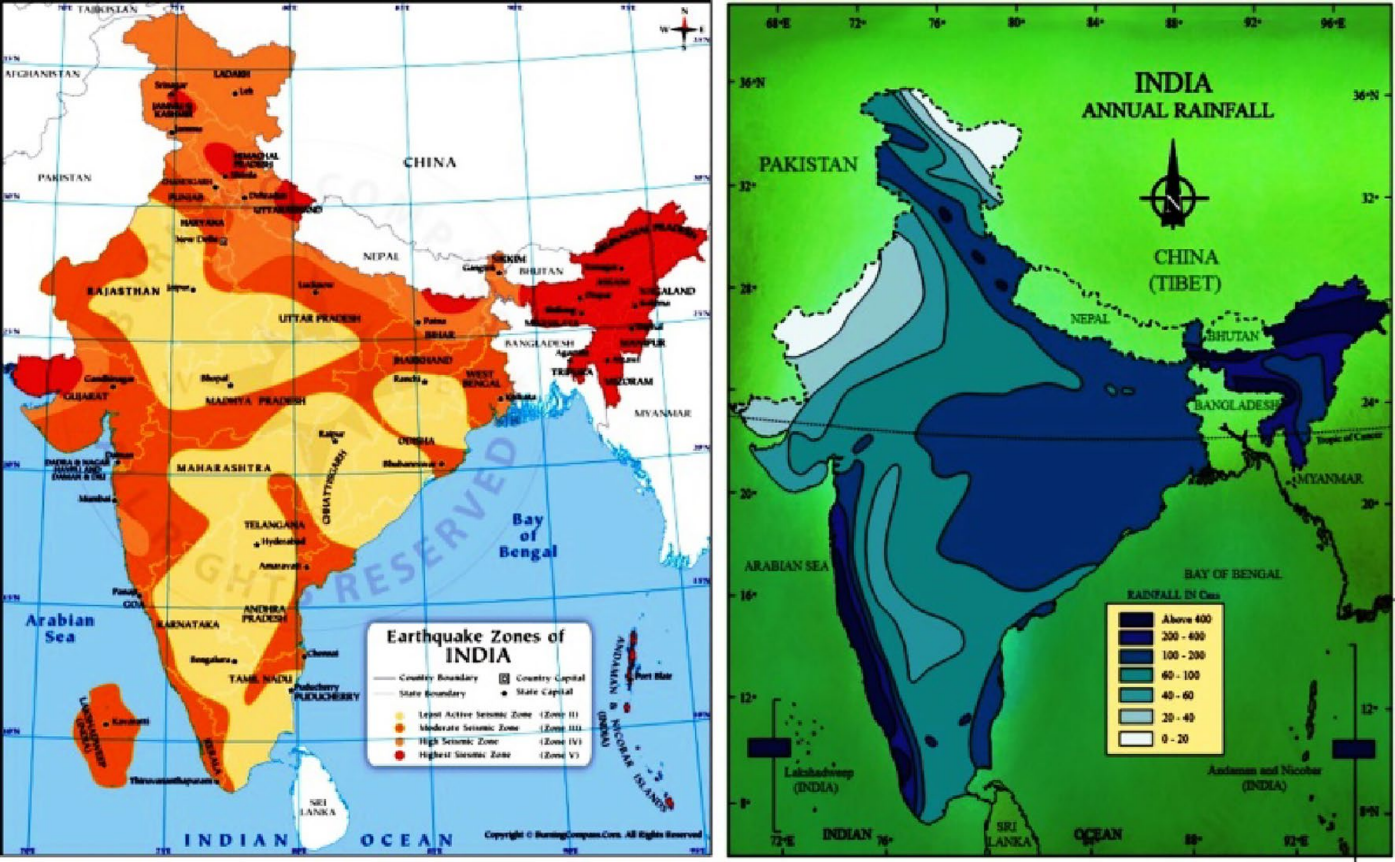
(**a**) Seismic zonation map of India. (**b**) Annual rainfall map of India.

**Figure 5:**
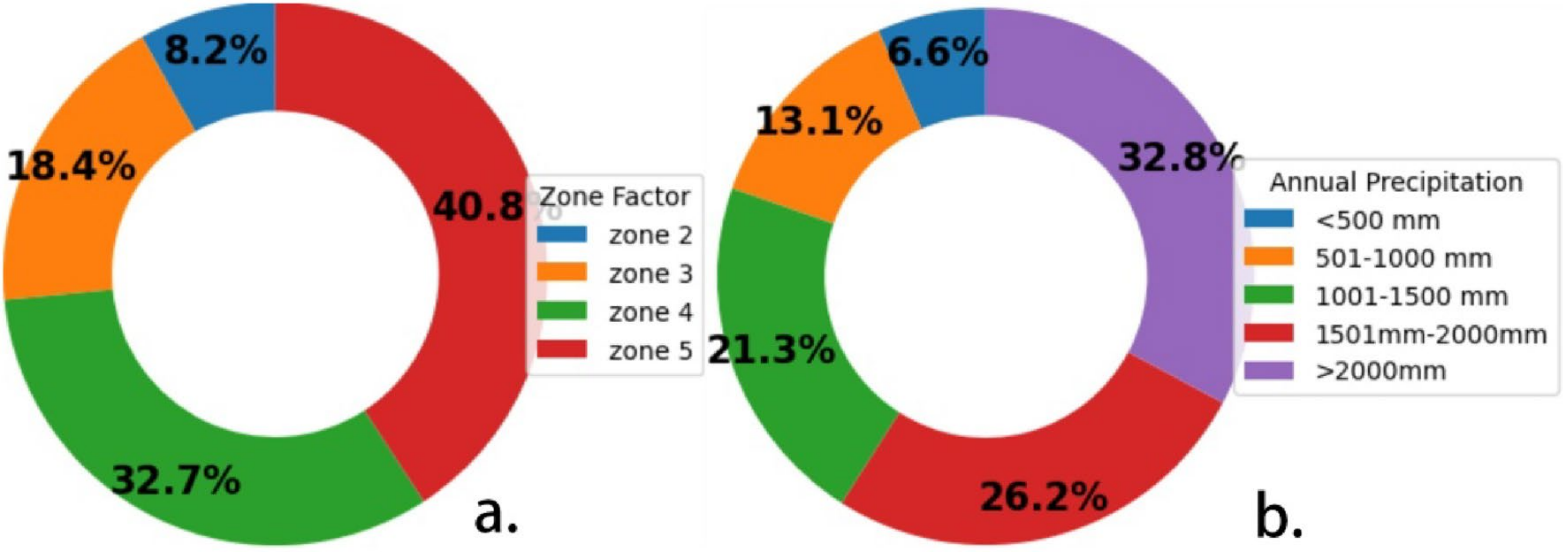
Percentage of Influence on slope instability (**a**). Seismic Zone (**b**). Annual precipitation.

The stability of rock slopes cannot be free from the adverse dynamic effects of earthquakes. The field observations have shown that earthquake-induced landslides are often associated with earthquakes of magnitude four or more Parkash^50^. If the ground is saturated with water, particularly following heavy rainfall, the shaking will result in more landslides than usual, with the volume of unstable material reaching several million m^3^. The investigation of slope and dam behaviour under seismic loading is a crucial and imperative issue. A flawed understanding of how slope and dams respond to seismic conditions can lead to irreversible damage Cai et al.^51^, Gordan et al.^52^. India has been subdivided into four seismic zones based on seismic intensity, with minimum seismic intensity for Zone-II to maximum seismic intensity for Zone-V^53^. clause (6.4.2) has provided zone factors for four seismic zones of India. The Himalayan region mostly lies in Zone IV and Zone V. The score values increase with increasing seismic intensity or earthquake-induced acceleration, and the maximum score is given to seismic zone-V (Table 2). Figure 4a depicts the seismic zonation map of India, and Fig. 5a depicts the percentage of influence of seismic zone in terms of assigned scores in causing earthquake-induced slope instability.

#### Annual precipitation

Rainfall (particularly in areas with significant monsoonal precipitation) is a primary factor that can trigger slope instability by the sudden increase in pore water pressure, which reduces the effective stress. The existing rock mass classification systems only account for the impact of groundwater on rock-cutting stability and do not consider the detrimental effects of surface water. The annual rainfall of the Himalayas varies from less than 250 mm in the Leh region to more than 12,000 mm in Mawsynram (Meghalaya). In general, rainfall in the north-western Himalayas lies between 1000 and 2500 mm, while in the north-eastern Himalayas is more than 1800 mm. The amount of annual rainfall in an area has been divided into five categories for scoring purposes, with the score increasing as the amount of annual rainfall increases (Table 2). Figure 4b depicts the annual rainfall map of India, and Figure 5b depicts the percentage of influence of annual rainfall range in terms of assigned score in causing rainfall-induced slope instability. Equation 7 is used to compute the score of the triggering effect.

7 $$TE=Earthquake\,force\,score+Annual\,precipitation\,score$$

### Anthropogenic effect (AE)

In recent times, anthropogenic activities have become a significant factor in causing slope instability, particularly in road-cut slopes. The stability of a slope depends on the excavation method used for these activities. To address this issue, this new classification system has tried to provide the scoring values for anthropogenic activities based on five major excavation modes. The maximum score has been given to poor blasting mode as it generates many secondary weak zones, further decreasing rock slope strength (Table 2). Anthropogenic effect score can be derived through Eq. 8.

8 $$AE=Score\,of\,slope\,excavation\,method$$

## Field case studies-based validation

**Figure 6:**
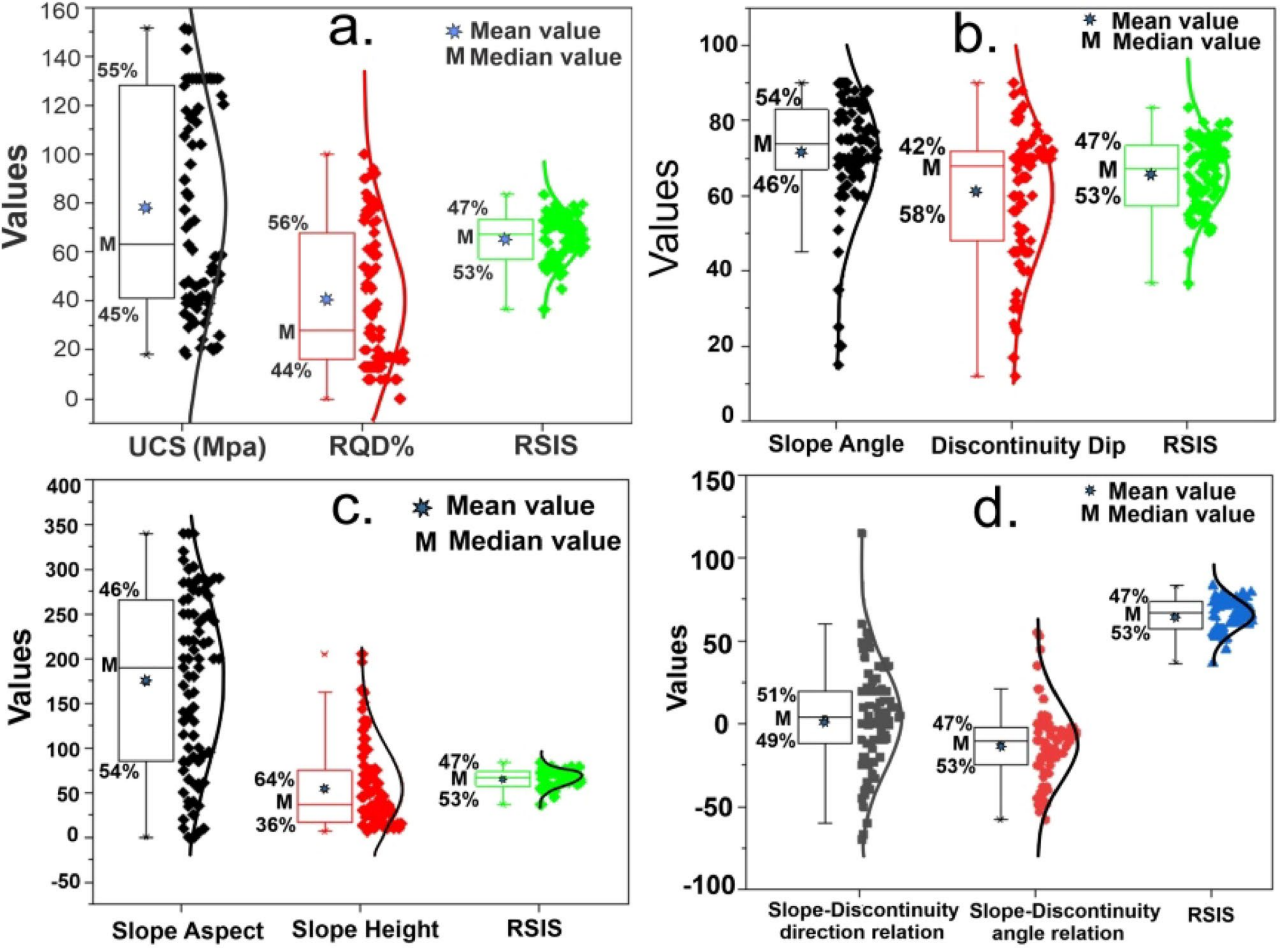
Box-and-whisker plots with scatter plots of RSIS with factors: (**a**) UCS and RQD, (**b**) Slope angle and discontinuity dip, (**c**) Slope aspect and slope height, (**d**) Slope-discontinuity direction and angle relation.

In order to assess the practical applications of the RSIS system, it is crucial to validate the newly developed classification system using an extensive number of case studies. For this purpose, 81 rock slopes from different locations across the Indian Himalayas were considered (Table 1). Various slopes were selected based on other geotechnical properties, lithologies, slope characteristics including geometric (slope height, slope angle & discontinuity dip), and different modes of excavation used. To provide more detailed information on the dataset used during the present study, visual representations in the form of box-and-whisker plots along with scatter plots, accompanied by the normal distribution function, statistical values, and comparison with RSIS, have been depicted in Fig. 6. It has been observed that slopes with low heights experience complete instability, while slopes with greater heights tend to exhibit a state of quasi-stability in RSIS classification. The occurrence of rainfall and earthquake greatly impacts the stability of rock slopes, and it is noteworthy that the case studies used for the validation in this research predominantly relates to areas with high rainfall and high seismicity, as all the slopes are located in the Himalayas.

The validation methodology adopted in this study involved the analysis of 53 case studies from the north-western and 28 case studies from the north-eastern part of the Himalayas. The details of validation parameters for all the case studies are shown in Table 5.

**Table 5:** Details of validation parameters for all the case studies.

Himalayan Region	State	No. of slopes	Seismic Zone	Variation range						
				Annual Rainfall	Strength Score	Slope angle	Slope height (m)	Slope Aspect	Lithology	Slope condition
Western	Jammu & Kashmir	14	Zone IV	1400 mm	4.5–15	48°–75°	75–205	10°–290°	Slate, Phyllite, Schist & Granitic Gniess	Completely Unstable (4); Unstable (9); Partially stable (1)
Western	Himachal Pradesh	10	Zone IV	2306 mm	6–9	68°–80°	45–120	130°–200°	Quartzite, Metabasic & Schict	Completely Unstable (3); Unstable (7);
Western	Uttarakhand	29	Zone IV & Zone V	1135 mm to 1800 mm	3–12	15°–90°	10–165	0°–340°	Limestone; Metavolcanic & Quartzite	Unstable(12); Partially stable (16); Stable (1)
Eastern	Sikkim	15	Zone IV	2200 mm	3–12	51°–87°	10–14	5°–303°	Phyllite, Gneiss & Quartzite	Completely Unstable (2); Unstable (8); Partially stable (4); Stable (1)
Eastern	Mizoram	13	Zone V	2500 mm	6–9	70°–90°	6–30	40°–340°	Shale & Sandstone	Completely Unstable (5); Unstable (8);

It is worth mentioning that the minimum score assigned to any slope as per the RSIS classification is 18.5, representing the stable class, while a score below 18.5 doesn’t exist. However, two slopes out of the validation case studies obtained scores below 45 (i.e., stable class), while the rest of the slopes fall in the active category of slope movement. Hence, they were found to be unstable in the field as well as in the RSIS classification system.

**Figure 7:**
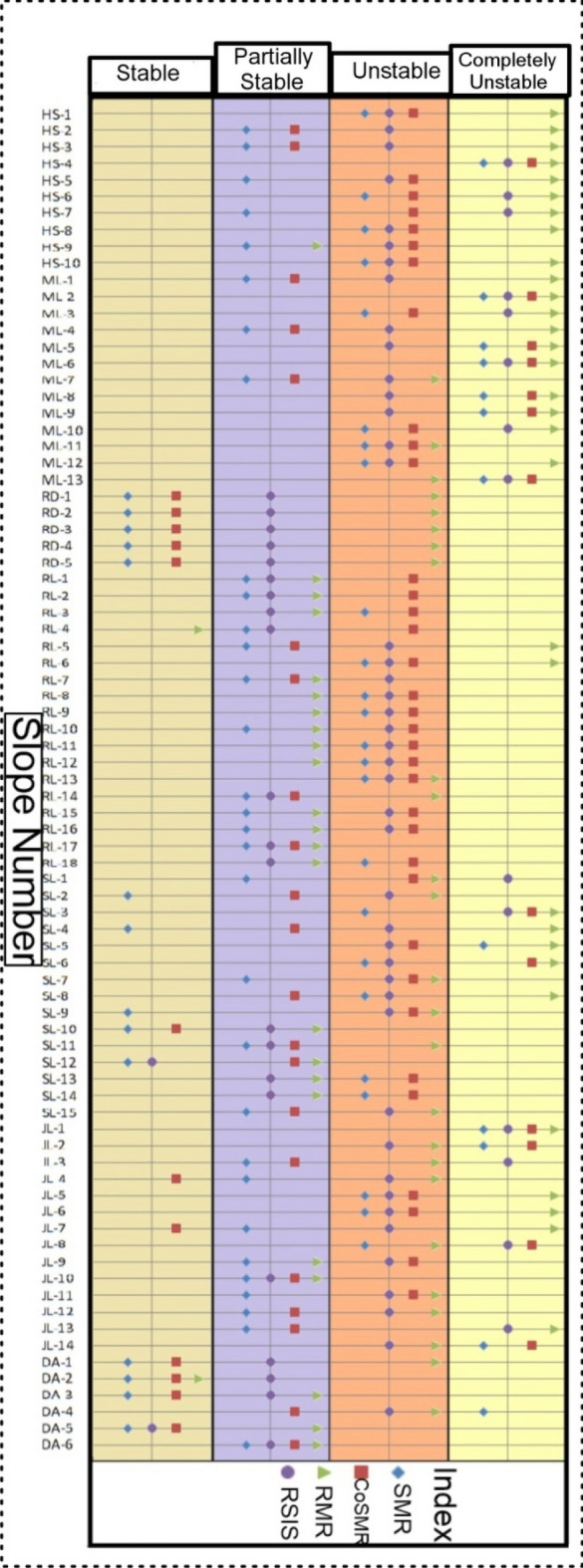
Slope wise comparative stability plots of RMR, SMR, CoSMR with RSIS for studied different rock slope.

Comparative analysis of the stability condition of slopes was conducted using the SMR and the CoSMR systems with the newly developed RSIS classification system. The results of this analysis are presented in Fig. 7. The comparison plot indicates that, in most cases, the RSIS system represents slope conditions more vulnerable than SMR and CoSMR. This vulnerability is attributed to the addition of triggering parameters such as annual rainfall and seismicity in the RSIS classification, which is a new addition to this classification system.

The RSIS system effectively expresses the influence of triggering parameters. Additionally, the RSIS system incorporates other factors, such as slope aspect and slope height, which also influence the stability of slope and have not been addressed in either SMR or CoSMR classification. The combined effect of these parameters will predict the slope condition more realistic and accurately as compared to SMR or CoSMR.

While it remains challenging for stability analysis methods to account for all potential parameters affecting rock slope stability, the proposed RSIS system incorporates a significant number of critical parameters. This enables a more accurate and precise analysis of slope conditions. The newly proposed classification system also comprehensively encompasses various structural parameters relevant to rock slopes, making it suitable for assessing the instability of rock slopes controlled by structural factors.

**Figure 8:**
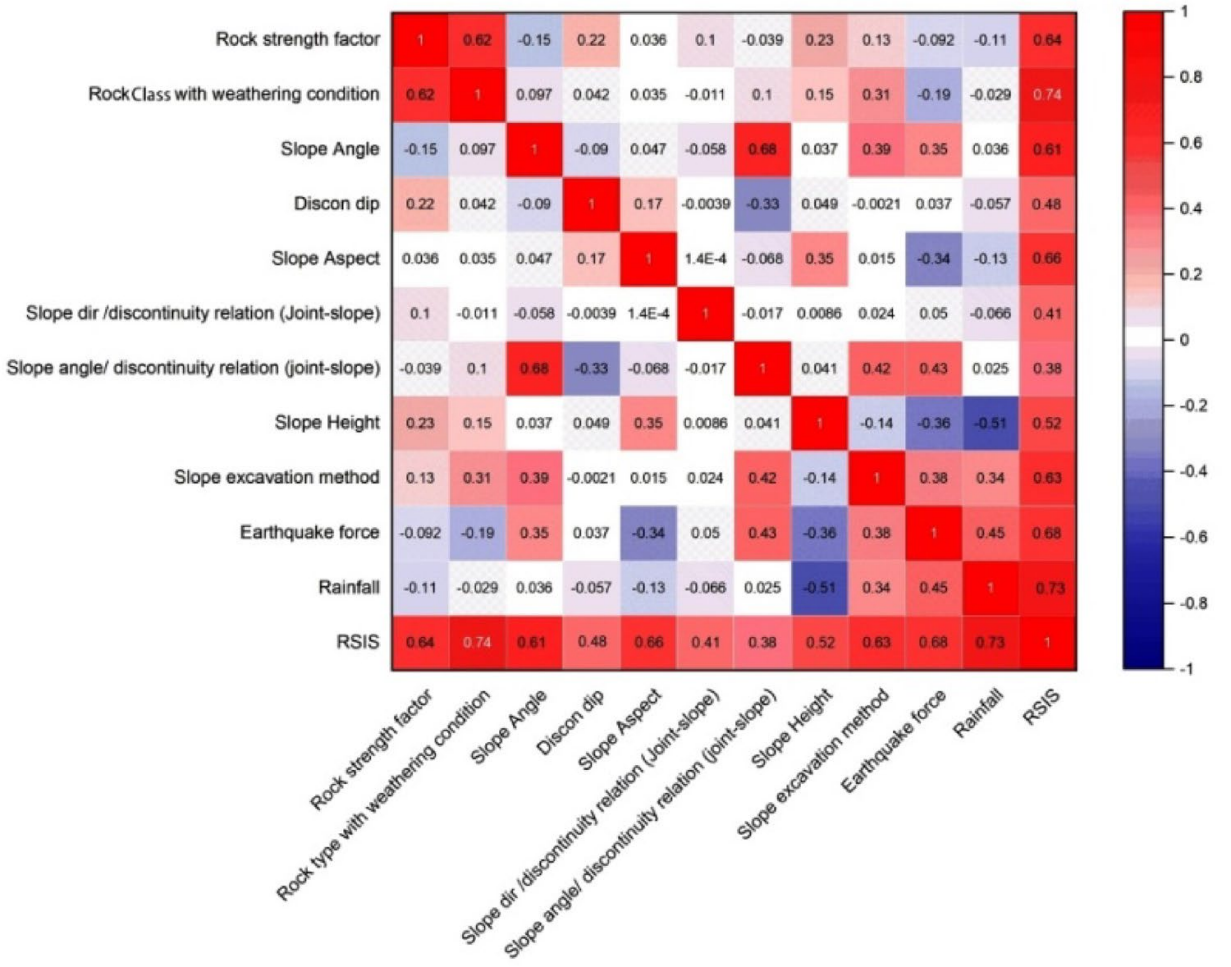
Correlations between eleven critical factors based on Pearson correlation coefficients.

In order to understand the simple correlation between RSIS and all the critical eleven parameters used in this new classification system, the Pearson correlation coefficient was computed. A Pearson coefficient value of 1 signifies the strongest positive correlation, whereas a coefficient value of − 1 indicates the strongest negative correlation. As shown in Fig. 8, the rock class with weathering conditions and rainfall exhibits a strong positive correlation with the Pearson correlation coefficient of 0.74 and 0.73, respectively. Additionally, the other factors such as slope angle, slope aspect, slope excavation methods, and seismic zone have also exhibited a strong positive correlation with Pearson correlation coefficient values of 0.61, 0.66, 0.63, and 0.68, respectively. Furthermore, factors including slope height, discontinuity dip, and the relationship between slope and discontinuity direction display a moderate relationship, as indicated by Pearson correlation coefficient values of 0.52, 0.48, and 0.41, respectively. However, the correlation between RSIS and the slope angle and discontinuity dip relation is notably weaker, with Pearson correlation coefficient values of 0.38.

**Figure 9:**
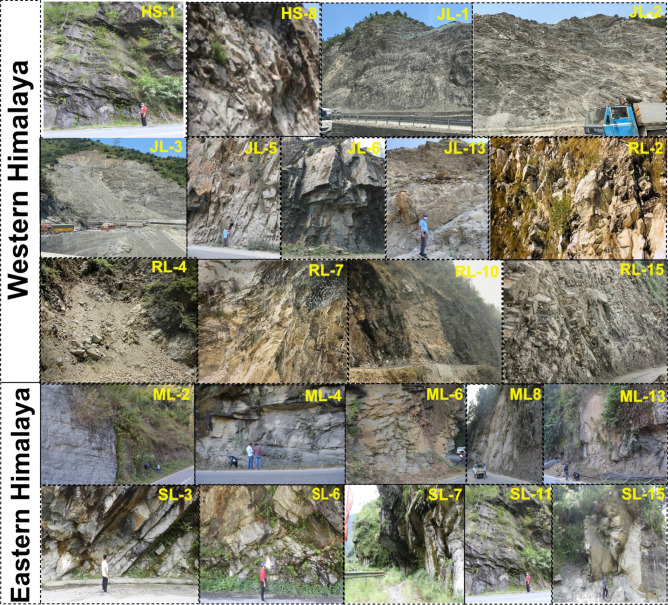
Field photographs illustrating the actual slope condition of field. The depicted case studies demonstrate the slopes to be in complete unstable condition. {“HS” Himachal Pradesh slope, “JL” Jammu & Kashmir slope, “RL” Rudraprayag slope, “ML” Mizoram slope, and “SL” Sikkim slope}.

Since all eleven parameters in the current developed classification system are derived from field observations, conducting field validation of the proposed RSIS system with the slope conditions is imperative. The field conditions of the majority of slopes at all the locations were found to be unstable, whereas few slopes in Mizoram, Sikkim, and Jammu & Kashmir were found to be critically stable, i.e., completely unstable. Field observation suggested that the conditions of most of the slopes were effectively assessed using the newly developed classification system. Field photographs of a few slopes from different study locations are shown in Fig. 9.

## Conclusion

The application of empirical classifications for the purpose of evaluating the stability condition of slopes has been used for many decades. Various classification systems have been devised for specific objectives, considering certain parameters like slope geometry, orientation of discontinuities, various parameters of discontinuities, and groundwater conditions. However, incorporating all possible parameters that influence the stability of rock slopes continues to be a challenging task. In recent years, the frequency and intensity of extreme weather conditions have increased due to climate change. The availability of advanced and sophisticated instruments enables the detection of even slight fluctuations in triggering parameters. These valuable information can be utilized in assessing the stability condition of slopes.

This study introduces a novel geological instability classification system, referred as RSIS, to evaluate the instability condition of rock slopes. This new system comprises four essential groups of parameters: rock characteristics, kinematic condition, triggering effect, and anthropogenic effect for assessing the condition of a rock slope. This new system integrates the rock strength factors along with parameters such as rock type (or lithology), slope angle and dip of discontinuities, the relationship between slope and discontinuities, and slope excavation method. The novel parameters proposed in this new RSIS classification system are slope height, slope aspect, annual precipitation, and seismic force have paramount importance for the design of rock slopes. These parameters have not been addressed or given due importance in earlier classification systems, while the current proposed system incorporates all these parameters for the first time. The instability score is derived through the summation of individual scores assigned to each of these parameters based on their field conditions. The addition of triggering and environmental parameters has produced accurate assessment. The sensitivity analyses have been investigated for all defined parameters of RSIS and the correlation coefficients have been examined and shown on the Pearson plot. Notably, the most of the parameters have demonstrated a robust positive correlation, with Pearson correlation coefficients between 0.74 and 0.61 while discontinuity dip, and the relationship between slope and discontinuity direction displayed a moderate relationship, as evidenced by Pearson correlation coefficient values of 0.48, and 0.41, respectively.

The relative weightage to each parameter was calibrated using data of 25 case studies from various sites in the Indian Himalayas.

Furthermore, the proposed system was validated and compared with the actual field condition of an additional 56 case studies of rock slopes from various states of the Indian Himalayas. Few scattering of scores obtained by RSIS exists, which is attributed to the limitations of field data collection. Also the primary limitation of the RSIS lies in its inability to assess slope conditions based on monthly or daily precipitation, which is crucial for real-time monitoring of rock slope stability. Additionally, the inadequate consideration of percentage deviations in defining the ranges of different parameters may have resulted in the inaccurate evaluation of certain slopes, a challenge that will be addressed in the future revised version of the RSIS system. The estimated score of the RSIS for most of the rock slopes are quite consistent with the actual slope condition and must be used with high confidence. The RSIS system proposed in this study will be quite useful tools for geologist and engineers involved in designing rock slopes.

## Data Availability

The datasets used and/or analysed during the current study available from the corresponding author on reasonable request.
