# Correction: A novel proposed classification system for rock slope stability assessment

**DOI:** 10.1038/s41598-026-39216-w

**Published:** 2026-02-24

**Authors:** Amit Jaiswal, A. K. Verma, T. N. Singh

**Affiliations:** Department of Civil and Environmental Engineering, Indian Institute of Technology Patna, Patna, 801106 India

Correction to: *Scientific Reports* 10.1038/s41598-024-58772-7, published online 14 May 2024

The original version of this Article contained an error in the legend of Figure 3, where a reference was omitted. The reference is listed below as Reference 43.

43. Pastor, J.L., Riquelme, A.J., Tomás, R. & Cano, M. Clarification of the slope mass rating parameters assisted by SMRTool, an open-source software. *Bull. Eng. Geol. Environ*. **78**(8), 6131–6142. (2019).

As a result,

“(**a**) Angular relationship between the strike of slope and strike of discontinuity (**b**) Angular relationship between slope angle and discontinuity dip.”

now reads:

“(**a**) Angular relationship between the strike of slope and strike of discontinuity (**b**) Angular relationship between slope angle and discontinuity dip. (*adopted after Pastor et al., 2019*^*43*^.)”

All subsequent References have been renumbered.

The original Article has been corrected.

